# Quality changes of duck meat during thermal sterilization processing caused by microwave, stepwise retort, and general retort heating

**DOI:** 10.3389/fnut.2022.1016942

**Published:** 2022-10-20

**Authors:** Xiaoqi Yang, Yan Li, Peng Wang, Donglei Luan, Jingxin Sun, Ming Huang, Baowei Wang, Yuandong Zheng

**Affiliations:** ^1^College of Food Science and Engineering, Qingdao Agricultural University, Qingdao, China; ^2^College of Food Science and Technology, Shanghai Ocean University, Shanghai, China; ^3^Qingdao Special Food Research Institute, Qingdao, China; ^4^National R&D Branch Center for Poultry Meat Processing Technology, Nanjing Huangjiaoshou Food Science and Technology Co., Ltd., Nanjing, China; ^5^Henan Province Qi County Yongda Food Co., Ltd., Hebi, China

**Keywords:** thermal sterilization, microwave, low-field NMR, Fourier transform infrared spectroscopy, amino acids, volatile flavor, microstructure

## Abstract

The quality changes of duck meat during thermal sterilization using microwave, stepwise retort and general retort heating were evaluated. Results showed that compared with stepwise retort and general retort, duck meat subjected to microwave showed significantly higher gumminess, chewiness, cohesiveness and resilience as well as glutamic acid, lysine and total amino acids. Low-field NMR revealed that the relative content of immobilized water after microwave and stepwise retort treatment was significantly higher than that after general retort treatment. The relative content of 1-octen-3-ol with characteristic mushroom aroma was significantly higher with microwave and stepwise retort heating than with general retort heating, while 2-pentyl-furan with poor taste was only detected with general retort heating. The muscle bundles subjected to microwave were neatly arranged, similar to those with no thermal sterilization. Overall, the meat quality after three thermal sterilization treatment was microwave > stepwise retort > general retort.

## Introduction

As meat is a perishable high-protein food, the choice of processing method is extremely important for prolonging the shelf life of meat products. Sterilization is the key to ensuring the safety and shelf life of meat products (1). Thermal sterilization is widely used for meat processing. However, various thermal sterilization techniques have different effects on the quality of meat products. Sterilization optimization minimizes changes in food quality and nutritional composition while ensuring commercial aseptic conditions. Previous studies have reported that commercial asepsis can be achieved when the F_0_ value (F_0_ defined as a thermal treatment that allows the reduction of 12 decimal reduction of *Clostridium botulinum* spores) reaches 3.0 min (2–4). However, thermal sterilization results in many uncontrolled physicochemical effects on food quality, regardless of the heat source power. General retort heating method decreases the quality of sterilized food owing to its low heat transfer rate and long sterilization time, which is unable to meet the demands of consumers for high-quality and high-nutrition food. Therefore, the food industry is committed to constant research and development of various new sterilization techniques to reduce quality loss and improve the quality of sterilized food (5).

In recent years, various new sterilization techniques have emerged in the food industry, such as irradiation sterilization (6), ohmic-assisted thermal sterilization (7), ZnO nanoparticles combined with radio frequency pasteurization (8), and microwave-assisted thermal sterilization (9). Among them, the microwave-assisted thermal sterilization technology has been approved by the U.S. Food and Drug Administration for the commercial sterilization of pre-packaged food, which ensures long-term storage of food at room temperature (9). The mechanism of microwave heating is that polar molecules rotate and collide under the action of microwave alternating electromagnetic field, which promotes the conversion of electromagnetic energy into thermal energy. In contrast to the traditional sterilization method of water bath heating, microwave heating can heat food from the inside, allowing the attainment of the target center temperature within a short time (10) and reducing the hot processing time. Related studies have reported that mashed potato and green pea model food treated with a microwave-assisted pasteurization system (915 MHz, 95°C) have lesser hot spot cooking values and color change than those subjected to traditional hot water treatment (95°C) (11). Moreover, Qu et al. (12) reported similar results that green beans treated with a microwave-assisted pasteurization system (915 MHz, 90°C) had lesser loss of chlorophyll A and ascorbic acid than those subjected to traditional hot water treatment (90°C). Variable temperature sterilization is a novel sterilization technology. Ansorena and Salvadori (13) found that the thiamine retention value of canned mussels treated with variable temperature (with 9 temperature stages between 110 and 140°C) was higher than that treated with constant temperature (134–137°C). Avila−Gaxiola et al. (14) determined a suitable processing temperature (75°C/19.0 min, 80°C/8.5 min, 90°C/10.7 min and 6°C/20.8 min) based on the vitamin C content, consistency index and color of canned papaya puree. In contrast to general retort heating, variable temperature heating is equivalent to stepwise retort heating, which can reduce the temperature difference between the food surface and the inside. Variable temperature heating not only guarantees adequate shelf life but also attenuates the effects of long-term high temperature on meat quality (15).

Although microwave and stepwise retort heating (variable temperature heating) methods have been studied in thermal sterilization with significant effects, the quality of meat has not yet been reported. Duck meat has the advantages of high nutrition, low fat, low cholesterol and high protein, and is favored by consumers (16). In this study, we investigated the effects of microwave, stepwise retort and general retort processing on duck meat quality. The comparison among the three thermal sterilization technologies is aimed at evaluating their application merits and demerits to not only ensure the F_0_ value of germicidal efficacy but also minimize negative impacts on meat quality.

## Materials and methods

### Materials

Fresh skinless Cherry Valley duck breast (pectoralis major, after rigor mortis) was purchased from New Hope Liuhe Co., Ltd. (Qingdao, China). All chemical reagents were of analytical or guaranteed purity.

### Preparation of thermally sterilized duck breast meat

#### No thermal sterilization (control)

Fresh skinless duck breast meat (10 cm × 5 cm × 1 cm) without any seasoning was heated in an 80°C water bath until the core temperature reached 70°C, and then vacuum-packed in polypropylene bags.

#### Microwave processing

**FIGURE 1:**
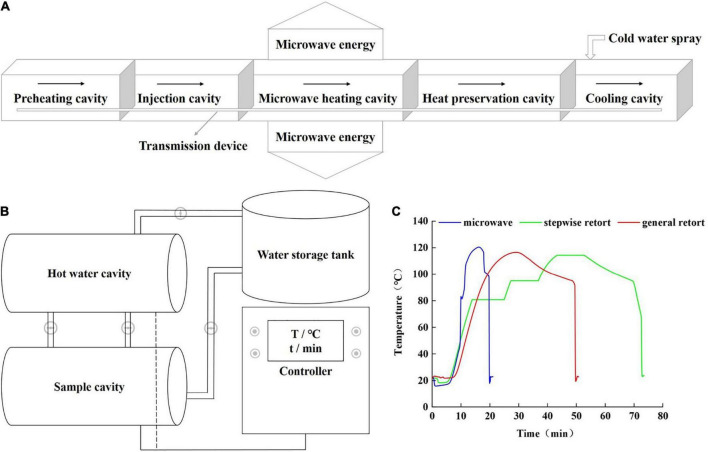
Schematic diagram of microwave treatment **(A)**, schematic diagram of stepwise retort and general retort treatments **(B)**, and temperature curves monitored by the apparatuses at the cold point of the samples **(C)**. Stepwise retort heating and general retort heating were performed at a progressive three-temperature (80–95–115°C) and a constant temperature (121°C) respectively.

The samples from section “No thermal sterilization (control)” were sterilized using an 896 MHz single mode microwave sterilization system (Shanghai, China) according to the method of Guo et al. (17). The microwave equipment was independently developed by Shanghai Ocean University. A schematic diagram of the equipment is shown in Figure 1A. The net power of the microwave source was 7 kW, and the sterilization process consisted of four steps: preheating, microwave heating, heat preservation and cooling. First, the samples were preheated to approximately 40°C in the preheating cavity and then passed through the injection cavity, microwave heating cavity, and heat preservation cavity filled with 121°C hot water at a pressure of 0.1 MPa at a specific speed and time through the transmission device. Finally, the temperature was reduced by spraying cold water through the cooling cavity. The total treatment time was 21.06 min, which included 9.2 min of preheating time, 2.4 min of microwave heating time, 4.63 min of holding time, and 4.83 min of cooling time.

#### Stepwise retort processing

The samples from section “No thermal sterilization (control)” were sterilized using an automatic water bath sterilizer (YT700; Zhucheng Yongtai Machinery Co., Ltd., China). A schematic diagram of the equipment is shown in Figure 1B. In the stepwise retort treatment, the samples were placed in the sample cavity, and automatic heating programs of 80 ± 1°C, 95 ± 1°C and 115 ± 1°C were set on the controller. Aconstant 15 min was maintained in each stage, and the pressure in the pot was 0.1 MPa. At the beginning of sterilization, the valve between the hot water cavity and sample cavity was opened, and the samples were sprayed with hot water. This process used a circulating pump to circulate hot water. After sterilization, the hot water in the sample cavity was recovered into the hot water cavity, and the cold water in the water storage tank entered the sample cavity to cool the samples. The total treatment time was 73.43 min.

#### General retort processing

The general retort treatment used the same equipment as the stepwise retort treatment; however, the program was different. The general retort treatment adopted one-step heating, with a sterilization temperature of 121°C, sterilization time of 9 min, pot pressure of 0.1 MPa, and the same sterilization principle as that described in section “Stepwise retort processing.” The total treatment time was 50.8 min.

#### Thermal lethality curve and F_0_ value

A wireless temperature sensor (PICO VACQ; TMI-ORION, France) was used to monitor the temperature change at the cold point of the samples; the temperature change curves are shown in Figure 1C. The F_0_ value of the samples were calculated using formula (1), and the F_0_ values of the microwave, stepwise retort and general retort treatments all reached 3.0 min, which was regarded as achieving the same sterilization effect and commercial sterility (2–4). The parameters treated by microwave, stepwise retort and general retort are the corresponding parameters with F_0_ value of 3.0 min obtained by many experiments.


(1) $$F_{0}=\int_{0}^{t}10^{\frac{T-T_{r\,e\,f}}{Z}}\,dt$$

where T is the cold point temperature at time t in the treatment process; T_ref_ is the reference temperature (121°C); and z is the target microorganism in meat products. The *z*-value of *Clostridium botulinum* is 10°C.

### Moisture content, pH value and shear force

The duck breast meat samples were crushed using a meat grinder (JS39D-250; Zhejiang Supor Co., Ltd., China) and placed in a rapid moisture tester (LGD-805A; Kunshan Lugong Precision Instrument Co., Ltd., China) to record the readings as expressed in % (w/w).

Each group of meat samples (5 g) was stirred and mixed with 50 mL of 0.1 mol/L KCl solution. A portable pH meter (STARTER300; OHAUS, USA) was directly inserted into the sample solution, and readings were recorded.

According to the method of Huo et al. (18), meat pieces with a size of 2 cm × 1 cm × 1 cm were cut along the direction parallel to the muscle fiber, and a piece of meat was placed in the groove of the digital muscle tenderness meter (C-LM3B; Tenovo, China) so that the muscle fiber was perpendicular to the edge direction; then, the maximum shear force in the process of cutting force was measured.

### Color

The inner surface of the meat samples was selected to determine lightness (L*), redness (a*) and yellowness (b*) using a colorimeter (CR-400; Konica Minolta Holdings Co., Ltd., Japan). Before the determination of these parameters, the device was corrected using a standard whiteboard.

### Textural properties

Textural properties of the meat samples were determined using a texture analyzer (TA.XT PlusC; Stable Micro Systems, UK). A P/36R probe was used in TPA mode, with a pre-measurement speed of 1.0 mm/s, middle-measurement speed of 1.0 mm/s, post-measurement speed of 5.0 mm/s, displacement of 5.0 mm, and trigger force of 5.0 g.

### Low field-nuclear magnetic resonance

Meat pieces with a size of 1 cm × 1 cm × 1 cm were cut into a 15 mm tube, which was placed into an NMR analyzer (NMI20-040V-I; Suzhou Niumag Analytical Instrument Corporation, China), and the transverse relaxation time (T_2_) was tested with a CPMG pulse sequence according to the method of Xu et al. (8) with slight modifications. The parameters were set as SW = 200 kHz, P1 = 8 μs, P2 = 16 μs, RG1 = 20 db, DRG1 = 3, TW = 3,000 ms, NS = 4, TE = 0.3 ms, and NECH = 6,000.

### Fourier transform infrared spectroscopy

The meat samples were freeze-dried using a vacuum freeze-drying machine (Pilot3-6E; Beijing Boyikang Experimental Instrument Co., Ltd., China) for 72 h and then ground to a powder. The atmosphere was used as a blank collection background, and a Fourier transform infrared spectrometer (Nicolet islo; Thermo Fisher Scientific, USA) was used for spectral scanning in the range of 4,000−400 cm^–1^ with a resolution of 4 cm^–1^ and a scanning frequency of 64 times.

### Amino acids

Using the method of Jo et al. (19), 50 mg of meat sample powder (see section “Fourier transform infrared spectroscopy”) was accurately weighed in a hydrolysis tube, added to 18 mL of 6 mol/L hydrochloric acid solution, and hydrolyzed for 22 h at 110 ± 1°C. The hydrolysate was transferred into a 50-mL apacity bottle. The volume of the hydrolysate was fixed with ultra-pure water, and the hydrolysate was dried under vacuum. The hydrochloric acid solution (0.02 mol/L) of 1 mL was accurately added to the hydrolysate, fully dissolved, filtered by 0.22 μm aqueous phase filter membrane, and the relative content of hydrolyzed amino acids in the samples was determined using an automatic amino acid analyzer (L-8900; Hitachi, Japan).

### Volatile flavor substances

The volatile flavor substances in the meat samples were detected using headspace solid-phase microextraction and gas chromatography-mass spectrometry (GC-MS) (TSQ8000; Samufei Co., Ltd., USA). The meat sample powder (see “Fourier transform infrared spectroscopy”) was placed in a solid phase microextraction bottle, and the extraction head was aged for 60 min (280°C) at the GC injection port, then inserted into the headspace part of the bottle, and extracted for 60 min at 60°C. After adsorption, the extraction head was removed and then desorbed at the injection port of the GC at 250°C for 2 min. The HP-5-MS capillary column (30 m × 0.25 mm, 0.25 μm) was used. The initial column temperature was 40°C, maintained for 3 min. After that, the temperature was raised to 200°C at 5°C/min, then to 240°C at 10°C/min, and retained for 10 min. The total running time was 49 min; the detection temperature was 240°C; the carrier gas was helium; the flow rate was 1.6 mL/min; the constant pressure was 13.02 kPa; the ion source temperature was 240°C; and the electron energy was 70 eV. *N*-alkanes C7-C30 were used to calculate the kovats index (KI) of each volatile flavor compound. The KI of volatile flavor compounds were calculated using formula (2). The compounds were searched using a computer and matched with the mainlib database. The positive and negative matching degrees were greater than 700 as a qualitative result.


(2) $$\text{KI}\,\left(\text{X}\right)=100*\text{Z}+100*\frac{\text{RT}\,\left(\text{X}\right)-\text{R}\,T\,\left(Z\right)}{\text{RT}\,\left(\text{Z}+1\right)-\text{R}\,T\,\left(Z\right)}$$

where KI(X) is the kovats index of the tested compound; RT(X) is the retention time of the tested compound; RT(Z) is the retention time of *n*-alkanes with carbon number Z; RT(Z+1) is the retention time of *n*-alkanes with carbon number Z+1. Among them, RT(X) is between RT(Z) and RT(Z+1).

### Scanning electron microscopy

The meat samples (0.5 cm × 0.5 cm × 0.5 cm) were added into 2.5% glutaraldehyde solution, fixed overnight at 4°C, and rinsed with phosphate buffer solution for six times (20 min/time). The rinsed meat samples were gradient dehydrated for 15 min with 50, 60, 70, 80, and 90% ethanol and dehydrated with anhydrous ethanol three times (30 min per time). After dehydration, the samples were replaced with tert-butanol three times (30 min each). The dehydrated meat samples were freeze-dried in vacuum (Pilot3-6E; Beijing Boyikang Experimental Instrument Co., Ltd., China) for 72 h, sprayed with gold using a vacuum ion sputtering coating machine, and observed using a scanning electron microscope (JSM-7500F; Japan Electronics, China).

### Statistics

All experiments were performed in triplicates. The significance (*P* < 0.05) of the experimental data was analyzed using one-way analysis of variance with the SPSS 26.0 software (IBM, USA). Data were expressed as mean ± standard deviation. Experimental charts were created using the ORIGIN 2019 software (Northampton, MA, USA).

## Results and discussion

### Moisture content, pH and shear force

Table 1 shows the effects of different treatments on the basic physiochemical properties of duck meat. The results showed no significant difference (*P* > 0.05) in the moisture content of meat before and after thermal sterilization, indicating that sterilization had little effect on the moisture content of meat. It is possible that vacuum packaging reduces water loss during sterilization of meat samples. Sterilization changes the proteins structure. Studies have shown that the increase in pH of cooked meat is due to the loss of acidic amino groups, exposure of basic amino residues and/or the formation of free hydrogen sulfide (20). The pH of meat in sterilization group was significantly higher than that in the control group (*P* < 0.05). For the sterilization groups, the order of the pH of meat was represented as general retort > stepwise retort > microwave. This indicated that microwave and stepwise retort methods can reduce the destruction of chemical bonds in protein structure, and this difference may be caused by the various heating intensities that produced various temperature effects on meat (8). In terms of shear force, the shear force was negatively correlated with meat tenderness. The shear force in sterilization groups was lower than that in the control group (*P* < 0.05), which may be due to the breaking of hydrogen bonds and disulfide bonds between proteins as well as the dissolution of collagen at high temperature. This indicated that sterilization aggravated the destruction of protein structure, but improved muscle tenderness to a certain extent (21). The shear force of microwave processing was significantly higher than that of general retort processing (*P* < 0.05), and there was no significant difference (*P* > 0.05) with stepwise retort processing. Therefore, the tenderness of duck meat was higher with general retort.

**TABLE 1:** Basic physiochemical properties of duck meat treated with no thermal sterilization and three sterilization strategies.

Items	Control	Microwave	Stepwise retort	General retort
Moisture content (%)	62.72 ± 1.05^a^	62.66 ± 0.62^a^	62.58 ± 0.82^a^	61.95 ± 1.45^a^
pH	6.13 ± 0.04^c^	6.20 ± 0.01^b^	6.23 ± 0.01^ab^	6.26 ± 0.02^a^
Shear force (N)	39.31 ± 0.83^a^	37.96 ± 0.40^b^	37.06 ± 0.49^bc^	36.33 ± 0.36^c^
L*	63.55 ± 0.47^a^	61.08 ± 0.81^b^	62.05 ± 0.74^b^	62.20 ± 0.48^b^
a*	6.94 ± 0.32^c^	9.09 ± 0.29^a^	8.45 ± 0.14^b^	8.72 ± 0.25^ab^
b*	9.10 ± 0.39^c^	10.93 ± 0.11^a^	10.45 ± 0.20^b^	10.61 ± 0.17^ab^
Hardness (g)	9539.71 ± 303.17^a^	8049.37 ± 255.80^b^	7507.55 ± 332.98^bc^	7111.46 ± 423.57^c^
Gumminess	3644.94 ± 55.85^a^	2207.76 ± 47.95^b^	2057.41 ± 57.27^c^	1957.85 ± 55.28^c^
Chewiness	1032.02 ± 48.82^a^	953.20 ± 23.83^b^	851.21 ± 35.13^c^	811.25 ± 18.60^c^
Springiness	0.34 ± 0.02^a^	0.32 ± 0.01^ab^	0.30 ± 0.02^bc^	0.29 ± 0.01^c^
Cohesiveness	0.39 ± 0.01^a^	0.35 ± 0.01^b^	0.33 ± 0.00^c^	0.30 ± 0.01^d^
Resilience	0.13 ± 0.00^a^	0.12 ± 0.00^b^	0.12 ± 0.00^c^	0.10 ± 0.00^d^

The values are expressed as mean ± standard deviation (*n* = 3). Different lowercase letters (a–d) in the same line represent significant differences (*P* < 0.05). L*, lightness; a*, redness; b*, yellowness.

### Color

Meat color is an important index for evaluating meat quality. The color difference of meat with different treatments is shown in Table 1. The L* values of microwave, stepwise retort and general retort processing methods were significantly lower after sterilization (*P* < 0.05), and there was no significant difference (*P* > 0.05) in the L* values of the three sterilization groups. After sterilization, the a* and b* values of duck breast meat increased significantly (*P* < 0.05), and the increasing trend was the same in all groups. The increasing degree of a * and b * values in the three sterilized groups was expressed as microwave > general retort > stepwise retort. The color change in duck breast meat may be related to the production of metmyoglobin by oxidative denaturation of myoglobin. Myoglobin and hemoglobin formed brown precipitates in meat (22), which resulted in a decrease in the L* value and an increase in the a* and b* values in duck meat. Compared with stepwise retort and general retort processing, microwave processing had lower L* values and higher a* and b* values, which may be related to microwave radiation. Microwave heating was to guide the rotation and collision of polar molecules such as water and ions by microwave electromagnetic field, which promoted the absorption of microwave energy on the surface of duck meat and converted it into heat energy, making it rapidly heat up (10). The rapid heating of microwave treatment accelerated the Maillard reaction of duck meat to produce brown or even black macromolecules, resulting in color changes (23). In addition, a higher a* value was a recognized result of microwave treatment of meat proteins (24).

### Textural properties

Textural properties are important for evaluating the quality of meat products, including hardness, springiness, cohesiveness, gumminess, chewiness and resilience. Sterilization of cooked meat products will further lead to changes in the structure of meat products and some temperature-related reactions will occur, resulting in the deterioration of related quality. Whereas different sterilization technologies result in different degrees of treatment and damage to the structure of meat products, thus having different effects on their textural characteristics (25).

The effect of different treatments on the textural properties of meat is displayed in Table 1. The results showed that the hardness, springiness, cohesiveness, gumminess, chewiness and resilience of meat decreased after sterilization. In the three sterilization processing, the hardness, springiness, cohesiveness, gumminess, chewiness and resilience of meat after microwave processing were significantly higher than that after general retort processing (*P* < 0.05). The cohesiveness and resilience of meat in general retort processing were lower than those in stepwise retort processing (*P* < 0.05). For microwave and stepwise retort processing, the gumminess, chewiness, cohesiveness and resilience of meat processed by microwave were significantly higher than those of stepwise retort (*P* < 0.05). The decrease in the textural characteristics of meat in sterilized groups may be owing to the increase in heat treatment degree, the further change in protein spatial structure, the decrease in myofibrillar protein cross-linking degree, and looseness of muscle state (26). This result was similar to previous results reported by Khan et al. (25) that heated and pressurized samples had lower hardness, gumminess, chewiness and higher tenderness, which was attributed to the difference between the pressure and heating medium in the exposure time. Wang et al. (27) showed that compared to water bath heating, microwave heating could induce more protein cross-linking and form a denser protein gel network structure. Generally, the textural characteristics of meat treated with microwave were better. In addition, the sensory quality of duck meat treated with different sterilization strategies was evaluated, and the scores of sensory evaluation of microwave and stepwise retort groups were higher (see Supplementary Table 1).

### Low field-nuclear magnetic resonance

**FIGURE 2:**
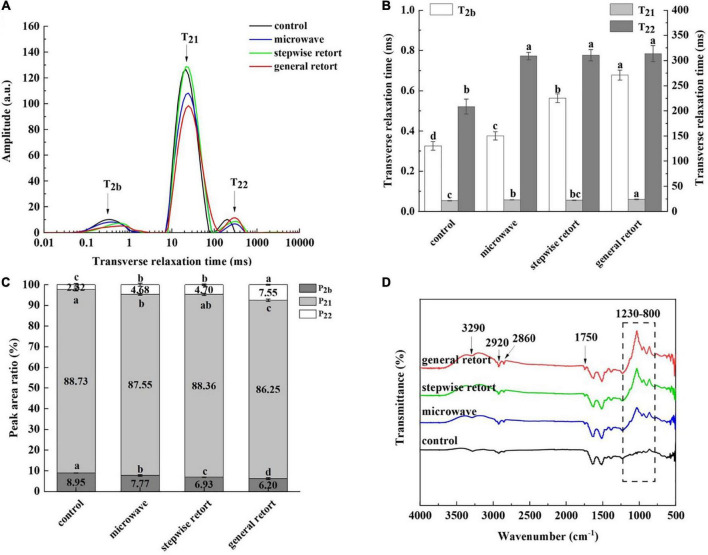
Distribution spectra **(A)**, transverse relaxation time T_2_ **(B)**, and peak area ratio **(C)** of water populations and FTIR spectroscopy analysis **(D)** for duck meat treated with no thermal sterilization and three sterilization strategies. Different lowercase letters (a–d) represent significant differences between different groups (*P* < 0.05).

Low field-nuclear magnetic resonance (LF-NMR) technology has been commonly used in the field of food processing because of its fast detection, small sample mass and non-destructive nature. The transverse relaxation time of LF-NMR was used to study the distribution and migration of water in meat products. Figure 2A shows the transverse relaxation time distribution of meat with different treatments. The water relaxation curves of meat subjected to different treatments had three peaks. There was a small peak at 0.1–10 ms that was generally believed to represent the water bound to macromolecules such as protein, which was described as bound water (T_2b_). A largest peak in the range of 10–100 ms was attributed to immobilized water (T_21_), which represented water in muscles that was difficult to flow between myofibrils and membranes. A small peak between 100 and 1,000 ms was generally attributed to free water (T_22_), which represented the flow of water in the intercellular space (28).

T_2_ represented the degree of binding between water and the matrix as well as the fluidity of water (29). The higher the T_2_ value, the looser the bond between water and the matrix and the greater the fluidity (30). As shown in Figure 2B, T_2b_ and T_22_ values in the sterilization groups were higher than those in the control group (*P* < 0.05), which demonstrated that the sterilization treatment decreased the binding degree of water to the matrix and increased the fluidity of water molecules in samples. T_2b_ and T_21_ values in general retort processing were higher than those in microwave and stepwise retort processing (*P* < 0.05). The T_2b_ value in stepwise retort processing was higher than that in microwave processing (*P* < 0.05), but there was no significant difference in T_21_ value (*P* > 0.05). For the value of T_22_, there was no significant difference between the sterilization groups (*P* > 0.05).

The ratio of peak area represented the relative content of different water populations. As shown in Figure 2C, immobilized water was the main form of moisture in matrix, and its peak area accounted for nearly 90% of the total peak area. The relative content of bound water and immobilized water was closely related to the water retention of meat. The higher the relative content, the stronger the water retention, and the quality of meat products will be better (8). P_2b_ value decreased and P_22_ value increased after sterilization (*P* < 0.05). The reason for this result may be that sterilization aggravated the destruction of protein network structure and reduced its binding ability to water; thus, the bound water and immobilized water in meat samples can be converted into free water more easily (31). The P_21_ value of general retort processing was lower than that of microwave and stepwise retort processing, whereas the P_22_ value was higher than that of microwave and stepwise retort processing (*P* < 0.05). This indicated that the relative contents of bound water and immobilized water in microwave and stepwise retort processing were higher than those in general retort processing (*P* < 0.05), and the relative content of free water was lower than that in general retort processing (*P* < 0.05). This was because the protein structure and the cell membrane of duck meat processed by microwave and stepwise retort was less damaged, and the network structure was denser, which can reduce the transfer of intracellular water to the intercellular space. On the other hand, the structural damage of duck protein treated by general retort was greater, which reduced the water-locking ability of duck protein, and made the immobilized water in the meat sample more easily converted into free water, which was consistent with the results of textural properties.

### Fourier transform infrared spectroscopy

Fourier transform infrared spectroscopy (FTIR) is a structural analysis based on the vibrations of functional groups and polar bonds in compounds. FTIR spectroscopy can be used to study the conformational changes in muscle proteins during meat processing. Figure 2D shows the FTIR spectra of meat with different treatments at a detection wavelength of 4,000–500 cm^–1^. The amide I band is related to the secondary structure of the protein, with a wavelength range is 1,700–1,600 cm^–1^, which represents C = O and a small amount of N-H stretching vibration (32). In this range, the peak intensity of the sterilization group was slightly higher than control, which may be due to the fact that the sterilization treatment destroyed the stability of the hydrogen bond to a greater extent. Thus, the helix structure was further unraveled, exposing hydrophobic residues and irregular random coil structure formed by the interaction between molecules (8).

Calabrò and Magazù (33) showed that methyl and methylene were observed in the 3,000–2,800 cm^–1^ C-H stretching vibration, and attributed the increase in methylene intensity to an increase in lipids caused by the Maillard reaction. Figure 2D shows that there were different intensity peaks at 2,860 and 2,920 cm^–1^ in each group; the peak intensity in control was the lowest, and the peak intensity in general retort processing was higher than that in microwave and stepwise retort processing, indicating that the degree of lipid oxidation in general retort processing was the highest. The spectra showed different trends at 3,500–3,000 cm^–1^, which may be caused by the C-H stretching vibration and N-H stretching vibration of the unsaturated carbon. The spectra of sterilization groups showed a small peak at 1,750 cm^–1^, which was related to the stretching vibration of C = O functional group and represented the content of triglyceride (34). The Maillard reaction is accompanied by the formation of initial intermediates, including the products of Amadori and Heyn’s, which contains CH_2_ and C = O compounds (35). In the range of 1,230–800 cm^–1^, strong peaks were observed in the microwave, stepwise retort and general retort processes. In particular, the peak of general retort processing was the strongest. This may be due to the asymmetric stretching of aliphatic phosphorus compounds containing P-O-C bonds, which may be related to the degree of lipid oxidation (33). Overall, the peak of general retort was the strongest, indicating that general retort processing had the greatest effect on the conformational changes of muscle protein and lipid.

### Amino acids

The type and relative content of amino acids in meat products determine the nutritional value and flavor of meat products, which is an important index for evaluating the quality of meat products. Generally high temperature promotes fat oxidation although it can give meat products a special aroma. Meanwhile, high temperature will cause a certain degree of damage to the nutritional value of meat products, including the degradation of amino acids (6). Table 2 shows the relative contents of hydrolyzed amino acids in meat treated with different treatments, and a total of 17 amino acids were detected, including 7 essential amino acids and 10 non-essential amino acids. The results showed that the relative content of total amino acids in sterilized groups was significantly lower than that in the control (*P* < 0.05), and the relative content of total amino acids of stepwise retort and general retort processing was lower than microwave processing (*P* < 0.05), revealing that the retention rate of amino acids in meat products sterilized by microwave was higher than other sterilization techniques.

**TABLE 2:** Hydrolyzed amino acids of duck meat treated with no thermal sterilization and three sterilization strategies.

Hydrolyzed amino acids	Relative content (%)			
	Control	Microwave	Stepwise retort	General retort
Aspartic acid	9.43 ± 0.06^a^	9.27 ± 0.07^ab^	8.97 ± 0.15^bc^	8.83 ± 0.26^c^
Threonine	4.78 ± 0.03^a^	4.64 ± 0.01^bc^	4.65 ± 0.03^b^	4.58 ± 0.05^c^
Serine	3.87 ± 0.04^a^	3.81 ± 0.07^a^	3.89 ± 0.02^a^	3.81 ± 0.12^a^
Glutamic acid	18.01 ± 0.15^a^	17.49 ± 0.03^b^	16.80 ± 0.08^c^	16.69 ± 0.34^c^
Glycine	3.95 ± 0.02^b^	4.01 ± 0.02^b^	4.03 ± 0.21^b^	4.67 ± 0.44^a^
Alanine	6.38 ± 0.06^a^	6.40 ± 0.09^a^	6.40 ± 1.15^a^	4.79 ± 0.30^b^
Cystine	1.99 ± 0.01^a^	1.86 ± 0.03^a^	1.54 ± 0.14^b^	1.42 ± 0.10^b^
Valine	4.71 ± 0.03^a^	4.56 ± 0.07^a^	4.62 ± 0.24^a^	4.84 ± 0.11^a^
Methionine	2.19 ± 0.17^a^	2.29 ± 0.21^a^	1.57 ± 0.05^b^	1.56 ± 0.08^b^
Isoleucine	4.49 ± 0.02^ab^	4.26 ± 0.21^b^	4.36 ± 0.14^ab^	4.59 ± 0.15^a^
Leucine	8.40 ± 0.10^a^	8.01 ± 0.03^bc^	7.79 ± 0.10^c^	8.11 ± 0.28^ab^
Tyrosine	4.14 ± 0.01^a^	4.14 ± 0.07^a^	3.99 ± 0.09^b^	4.11 ± 0.07^ab^
Phenylalanine	4.42 ± 0.07^a^	4.36 ± 0.07^a^	3.88 ± 0.07^b^	3.97 ± 0.04^b^
Lysine	8.64 ± 0.06^a^	8.41 ± 0.06^b^	8.12 ± 0.07^c^	8.01 ± 0.12^c^
Histidine	2.72 ± 0.04^a^	2.58 ± 0.06^b^	2.73 ± 0.01^a^	2.76 ± 0.05^a^
Arginine	6.89 ± 0.12^a^	6.70 ± 0.06^a^	5.79 ± 0.41^b^	6.34 ± 0.35^ab^
Proline	3.20 ± 0.09^bc^	3.16 ± 0.07^c^	3.40 ± 0.07^ab^	3.53 ± 0.15^a^
EAAs	37.63 ± 0.25^a^	36.52 ± 0.29^b^	34.99 ± 0.59^c^	35.66 ± 0.54^bc^
NEAAs	60.58 ± 0.35^a^	59.43 ± 0.28^b^	57.53 ± 0.56^c^	56.95 ± 0.03^c^
TAAs	98.21 ± 0.57^a^	95.95 ± 0.05^b^	92.52 ± 0.27^c^	92.61 ± 0.57^c^

The values are expressed as mean ± standard deviation (*n* = 3). Different lowercase letters (a–c) in the same line represent significant differences (*P* < 0.05). EAAs, essential amino acids; NEAAs, non-essential amino acids; TAAs, total amino acids.

The relative contents of essential amino acids, non-essential amino acids and total amino acids in sterilized groups were lower than those in control (*P* < 0.05). For the sterilization groups, the relative content of essential amino acids after microwave processing was the highest and that after stepwise retort processing was the lowest. The relative content of most amino acids such as aspartic acid, threonine, glutamic acid, cystine, leucine, tyrosine, phenylalanine, lysine and arginine decreased in sterilized meat. The content of only a small number of amino acids such as glycine and alanine increased. The combination of aspartic acid, glutamic acid, tyrosine, phenylalanine, alanine and glycine with sodium salt will show a special fresh taste and give meat products a good taste. Glutamic acid was the main flavor amino acids, and its content directly affected the flavor of meat products. However, the relative content of glutamic acid decreased after sterilization (*P* < 0.05), thereby reducing the fresh taste of meat. Lysine and arginine are critical for the nutritional value of meat (2). Lysine is the first limiting amino acids in the human body that can compensate for the deficiency of lysine in cereal protein and ensure the dietary balance of the human body. Nonetheless, sterilization caused damage to these amino acids and reduced the nutritional value of meat. Glycine can synthesize glutathione and has antioxidant effects. In addition, histidine is the precursor of carnosine synthesis and has antioxidant effects (36). Generally, microwave processing resulted in higher amino acid retention rate and lower damage to nutrients than other meat sterilization techniques.

### Volatile flavor substances

The aroma of cooked meat products is an important feature that affects the sensory quality and consumer acceptance of meat products. Therefore, accurate detection of volatile flavor substances in different sterilized meat is of great significance to the processing and utilization of meat products. The volatile flavor compounds of meat treated with different sterilization methods were analyzed using GC-MS. As shown in Table 3, 83 compounds were identified and divided into seven categories: Hydrocarbons, alcohols, aldehydes, ketones, esters, acids and others. In the control, microwave, stepwise retort and general retort processing groups, 54, 56, 54, and 67 volatile compounds were detected, respectively. These compounds jointly affect the smell of meat, but the magnitude of the effect is determined by the threshold and relative content of these compounds (37).

**TABLE 3:** Volatile flavor substances of duck meat treated with no thermal sterilization and three sterilization strategies.

No.	Volatile compounds	KI	Relative content (%)			
			Control	Microwave	Stepwise retort	General retort
	**Hydrocarbons**					
1	Decane	977.34	0.10 ± 0.00			
2	Tetradecane, 2,6,10-trimethyl-	1194.08	1.33 ± 0.11^a^	0.70 ± 0.12^b^	1.58 ± 0.23^a^	0.76 ± 0.05^b^
3	Octadecane, 6-methyl-	993.48	0.15 ± 0.01			
4	Octane, 3,3-dimethyl-	996.60	0.60 ± 0.06			
5	Undecane	1068.88	4.86 ± 0.66^a^	3.85 ± 0.91^ab^	3.31 ± 0.60^b^	2.79 ± 0.03^b^
6	Dodecane, 4,6-dimethyl-	1411.15	1.25 ± 0.09			
7	Octane, 6-ethyl-2-methyl-	1080.66	0.27 ± 0.02			
8	2,3-Dimethyldecane	1128.99	0.71 ± 0.16^a^	0.46 ± 0.08^b^	0.56 ± 0.07^ab^	0.39 ± 0.03^b^
9	Dodecane	1163.19	3.11 ± 0.60^a^	2.19 ± 0.49^bc^	2.68 ± 0.24^ab^	1.48 ± 0.11^c^
10	Undecane, 2,6-dimethyl-	1174.59	0.91 ± 0.02			
11	Dodecane, 4-methyl-	1182.74	1.15 ± 0.19^a^	0.88 ± 0.10^a^	0.91 ± 0.20^a^	0.44 ± 0.09^b^
12	Tetradecane	1420.56	2.36 ± 0.18^a^	2.54 ± 0.42^a^	1.54 ± 0.33^b^	1.47 ± 0.14^b^
13	Hexadecane	1624.39	0.55 ± 0.10^b^	0.53 ± 0.10^b^	1.14 ± 0.14^a^	0.40 ± 0.01^b^
14	Dodecane, 2,6,11-trimethyl-	1457.49	5.34 ± 0.32^a^	3.42 ± 0.47^c^	4.73 ± 0.10^b^	0.52 ± 0.07^d^
15	2,6,10-Trimethyltridecane	1420.91	0.69 ± 0.09^ab^	0.21 ± 0.03^c^	0.87 ± 0.12^a^	0.56 ± 0.13^ab^
16	Tetradecane, 4-methyl-	1470.85	0.11 ± 0.04^b^	0.11 ± 0.03^b^	0.29 ± 0.14^a^	0.09 ± 0.03^b^
17	Non-adecane, 2-methyl-	1433.33	0.48 ± 0.03			
18	Eicosane	1634.55	0.16 ± 0.02			
19	Decane, 2,6,7-trimethyl-	997.45		0.40 ± 0.08^a^	0.45 ± 0.09^a^	0.26 ± 0.11^a^
20	Pentadecane	1433.10		2.19 ± 0.25^b^	2.97 ± 0.15^a^	0.58 ± 0.13^c^
21	Dodecane, 2,6,10-trimethyl-	1433.45		0.28 ± 0.07^b^	0.45 ± 0.06^b^	1.52 ± 0.40^a^
22	Heptadecane, 2,6,10,15-tetramethyl-	1433.33		0.53 ± 0.16^b^	0.61 ± 0.13^ab^	0.88 ± 0.11^a^
23	Heptadecane	1625.49	0.44 ± 0.11^a^	0.40 ± 0.10^a^	0.52 ± 0.12^a^	0.18 ± 0.05^b^
24	(E)-1-Phenyl-1-butene	1113.68	0.42 ± 0.09^a^	0.28 ± 0.07^b^	0.29 ± 0.01^ab^	0.18 ± 0.07^b^
25	Benzene, 2-ethenyl-1,3,5-trimethyl-	1408.71	0.24 ± 0.06^a^	0.20 ± 0.06^a^	0.21 ± 0.07^a^	0.15 ± 0.01^a^
26	3-Eicosene, (E)-	1438.43	0.09 ± 0.02^b^	0.22 ± 0.06^a^	0.12 ± 0.01^b^	0.13 ± 0.03^b^
27	3-Octyne, 7-methyl-	1008.46	0.48 ± 0.08^a^	0.47 ± 0.09^a^	0.30 ± 0.03^b^	0.30 ± 0.05^b^
28	Benzene, 1-ethyl-2,4-dimethyl-	1054.68	0.39 ± 0.05			
29	Benzene, 1,2,4,5-tetramethyl-	1087.31	2.98 ± 0.21^a^	1.67 ± 0.19^d^	2.41 ± 0.09^b^	2.06 ± 0.07^c^
30	Benzene, 1-methyl-4-(1-methylpropyl)-	1138.76	0.84 ± 0.08^a^	0.61 ± 0.08^b^	0.62 ± 0.03^b^	0.52 ± 0.03^b^
31	Benzene, 1-ethyl-3,5-dimethyl-	1115.96	1.63 ± 0.10^a^	1.65 ± 0.09^a^	1.55 ± 0.06^a^	1.60 ± 0.18^a^
32	Benzene, 1,4-diethyl-2-methyl-	1131.60	0.19 ± 0.01			
33	Benzene, 1-ethyl-2,4,5-trimethyl-	1158.96	0.88 ± 0.04			
34	Benzene, 1,3-dimethyl-5-(1-methylethyl)-	1192.18	0.40 ± 0.02			
35	Benzene, 2-ethyl-1,4-dimethyl-	1054.98		0.29 ± 0.09^a^	0.26 ± 0.03^a^	0.20 ± 0.02^a^
	Total		33.11 ± 0.73^a^	24.08 ± 1.09^c^	28.37 ± 0.74^b^	17.46 ± 0.53^d^
	**Alcohols**					
1	1-Octen-3-ol	964.87	3.83 ± 0.06^c^	5.13 ± 0.26^a^	4.71 ± 0.46^a^	4.15 ± 0.80^b^
2	1-Heptanol, 2-propyl-	1033.53	0.58 ± 0.12			
3	1-Octanol, 2-butyl-	1429.27	0.16 ± 0.03^b^	0.32 ± 0.08^a^	0.24 ± 0.07^ab^	0.10 ± 0.09^b^
4	2,6-Dimethyl-1-nonen-3-yn-5-ol	1419.93	7.15 ± 1.78^b^	14.98 ± 1.63^a^	11.88 ± 3.17^a^	16.40 ± 2.43^a^
5	Cyclopentadecanol	1554.62	0.16 ± 0.01			
6	1-Hexadecanol	1438.82		0.35 ± 0.04^a^	0.12 ± 0.03^b^	0.36 ± 0.10^a^
7	Ethanol, 2-(9-octadecenyloxy)-, (Z)-	1653.64		0.09 ± 0.05^a^	0.03 ± 0.03^a^	0.07 ± 0.04^a^
8	3-Nonen-2-ol, (Z)-	914.45		0.10 ± 0.06^b^	0.23 ± 0.05^a^	0.16 ± 0.04^ab^
9	6-Methyl-6-hepten-4-yn-3-ol	1008.76				0.44 ± 0.04
10	Carveol	1054.98				0.09 ± 0.02
11	1-Hexadecanol, 2-methyl-	1466.55				0.09 ± 0.00
	Total		11.88 ± 1.53^c^	20.97 ± 1.35^ab^	17.21 ± 3.66^b^	21.86 ± 3.06^a^
	**Aldehydes**					
1	Hexanal	771.43	4.15 ± 0.72^a^	3.51 ± 0.70^b^	3.55 ± 0.94^b^	2.80 ± 0.73^c^
2	Heptanal	893.02	0.36 ± 0.09^a^	0.27 ± 0.03^a^	0.31 ± 0.05^a^	0.24 ± 0.12^a^
3	2-Heptenal, (Z)-	945.04	0.27 ± 0.07^a^	0.19 ± 0.08^ab^	0.26 ± 0.09^a^	0.10 ± 0.05^b^
4	Benzaldehyde	948.73	0.31 ± 0.06^b^	0.31 ± 0.07^b^	0.46 ± 0.06^a^	0.48 ± 0.07^a^
5	Octanal	983.29	0.94 ± 0.21^a^	0.29 ± 0.23^b^	0.28 ± 0.15^b^	0.26 ± 0.08^b^
6	Non-anal	1075.83	5.40 ± 0.94^a^	2.37 ± 0.63^b^	3.48 ± 0.37^b^	2.31 ± 1.34^b^
7	Decanal	1170.68	1.38 ± 0.35^a^	0.81 ± 0.25^ab^	1.08 ± 0.48^ab^	0.41 ± 0.25^b^
8	2-Octenal, 2-butyl-	1425.83	0.42 ± 0.11^b^	1.73 ± 0.23^b^	0.57 ± 0.11^b^	4.62 ± 1.30^a^
9	Octadecanal	2029.10	0.26 ± 0.12^ab^	0.08 ± 0.05^b^	0.17 ± 0.14^ab^	0.29 ± 0.03^a^
10	2-Hexenal, 2-ethyl-	985.27		0.38 ± 0.10^b^	0.11 ± 0.03^c^	0.80 ± 0.16^a^
11	2-Octenal, (E)-	1034.14		0.89 ± 0.02^a^	1.00 ± 0.09^a^	0.87 ± 0.08^a^
12	10-Octadecenal	1403.69		0.08 ± 0.01^a^		0.04 ± 0.01^a^
13	Tridecanal	1458.43		0.10 ± 0.00		
14	Hexadecanal	2029.85		0.13 ± 0.01^a^	0.07 ± 0.03^b^	0.07 ± 0.01^b^
15	2-Butylhept-2-enal	1441.11				0.88 ± 0.03
16	Pentadecanal-	1458.82				0.08 ± 0.00
	Total		13.49 ± 2.72^a^	11.14 ± 0.30^a^	11.34 ± 1.48^a^	14.25 ± 0.80^a^
	**Ketones**					
1	2,5-Octanedione	967.42	15.26 ± 0.81^a^	11.93 ± 1.25^b^	10.52 ± 2.25^bc^	8.29 ± 0.51^bc^
2	3-Octen-2-one, (E)-	1014.80	4.46 ± 0.49			
3	3,5-Octadien-2-one, (E,E)-	1045.32	1.84 ± 0.25^b^	2.59 ± 0.40^b^	2.59 ± 0.68^b^	3.74 ± 0.44^a^
4	2-Non-anone	1063.75	0.58 ± 0.10^a^	0.52 ± 0.10^a^	0.55 ± 0.07^a^	0.28 ± 0.06^b^
5	Isophorone	1091.84	1.15 ± 0.16^a^	0.67 ± 0.11^b^	1.03 ± 0.11^a^	0.47 ± 0.10^b^
6	2,5-Piperazinedione, 3-methyl-	1151.47	2.55 ± 0.28^a^	0.70 ± 0.18^c^	2.21 ± 0.54^ab^	1.82 ± 0.15^b^
7	2-Decanone	1157.65	0.85 ± 0.06^a^	1.37 ± 0.12^a^	1.26 ± 0.73^a^	0.98 ± 0.21^a^
8	5,9-Undecadien-2-one, 6,10- dimethyl-, (E)-	1498.52	0.30 ± 0.08^a^	0.36 ± 0.07^a^	0.36 ± 0.04^a^	0.35 ± 0.08^a^
9	3-Octen-2-one	1015.11		6.83 ± 0.82^a^	6.94 ± 0.87^a^	8.22 ± 1.17^a^
10	Cyclodecanone	1499.30		0.40 ± 0.06^b^	0.35 ± 0.10^b^	1.28 ± 0.10^a^
11	1-Octen-3-one	961.47		0.14 ± 0.03^a^	0.23 ± 0.06^a^	0.23 ± 0.06^a^
12	3-Nonen-2-one	1108.14				0.29 ± 0.00
13	Ketone, 2,2-dimethylcyclohexyl methyl	1143.00				0.04 ± 0.01
14	4-Acetonylcycloheptanone	1436.93				0.20 ± 0.01
	Total		26.99 ± 3.15^a^	25.51 ± 3.21^a^	26.04 ± 3.20^a^	26.19 ± 2.33^a^
	**Esters**					
1	Diethyl phthalate	1534.03	0.12 ± 0.03^b^	0.22 ± 0.05^a^	0.28 ± 0.02^a^	0.23 ± 0.03^a^
2	Dibutyl phthalate	2096.27	0.44 ± 0.11^b^	1.19 ± 0.12^a^	1.10 ± 0.24^a^	0.13 ± 0.03^c^
3	2(3H)-Furanone, 5-hexyldihydro-	1151.47		2.61 ± 0.37^a^	0.74 ± 0.10^b^	0.59 ± 0.08^b^
	Total		0.56 ± 0.09^c^	4.02 ± 0.22^a^	2.12 ± 0.32^b^	0.95 ± 0.11^c^
	**Acids**					
1	Dodecanoic acid, 3-hydroxy-	1053.17				0.07 ± 0.00
2	10,12-Octadecadiynoic acid	1072.81				0.01 ± 0.00
	Total		0.00	0.00	0.00	0.08 ± 0.00
	**Other**					
1	Furan, 2-pentyl-	971.67				0.25 ± 0.05
2	1H-Pyrrole, 1-pentyl-	1174.92				1.15 ± 0.04
	Total		0.00	0.00	0.00	1.40 ± 0.05

The values are expressed as mean ± standard deviation (*n* = 3). Different lowercase letters (a–d) in the same line represent significant differences (*P* < 0.05).

After sterilization, the relative content of hydrocarbons in the volatile flavor compounds of meat decreased significantly, and the relative content of alcohols increased significantly (*P* < 0.05). Due to the high flavor threshold of hydrocarbons, they generally contribute little to the flavor of meat. In contrast, the flavor threshold of unsaturated alcohols was lower, which had a significant effect on the flavor of meat. Alcohols are generally produced by the breakdown of linoleic acid in muscles by lipoxygenase and peroxidase and mostly have a fresh and sweet taste (38). The relative content of 1-octen-3-ol and 2, 6-dimethyl-1-nonen-3-yn-5-ol in the alcohols was relatively high, especially in the sterilization groups, which was higher than that in the control (*P* < 0.05). Studies have shown that 1-octen-3-ol has a mushroom flavor, which is the characteristic aroma of meat products (39). The relative content of 1-octene-3-ol in microwave and stepwise retort processing was higher than that in general retort processing (*P* < 0.05).

Aldehydes are considered important odorous substances produced by oil oxidation. They are involved in the interaction between amino acids and carbonyl groups and are important intermediates in fat oxidation. The thresholds of aldehydes were lower than alcohols and they were the most important volatile flavor substances in meat products. Li et al. (40) showed that hexanal, heptanal, octanal and non-anal are produced by thermal oxidation or degradation of linoleic acid or linolenic acid. There was no significant difference (*P* > 0.05) in the relative content of heptanal, octanal and non-anal among the sterilized groups and the relative content of hexanal in general retort processing was lower than that in microwave and stepwise retort processing (*P* < 0.05). Benzaldehyde is produced *via* degradation of phenylalanine through the Strecker degradation reaction. The relative content of benzaldehyde in stepwise retort and general retort processing was higher than that in the control and microwave processing (*P* < 0.05), which would result in poor taste of meat products (38). The relative content of unsaturated aldehyde in the sterilized groups was higher, and (E)-2-octenal with fat and meat aroma was only detected in the sterilized groups. However, there was no significant difference in the relative content of (E)-2-octenal between the sterilized groups (*P* > 0.05). Xie et al. (41) revealed that unsaturated aldehydes are characteristic aroma compounds produced during the heating of animal fats. These compounds can be further oxidized to form furans, alcohols, and other compounds.

Sicuro (42) proved that ketones are secondary metabolites of peroxidation of unsaturated fatty acids, which have a creamy flavor and a positive effect on the flavor of meat products. The threshold of ketones was much higher than that of aldehydes, which played a coordinating role in the overall volatile flavor of meat products. The highest relative ketone content in all groups was 2, 5-octanedione, and the relative content of the control was significantly higher than that of the other groups (*P* < 0.05). The relative content of (E)-3-octen-2-one, (E,E)-3,5-octadien-2-one and 3-octen-2-one was also higher, in which (E)-3-octen-2-one was only detected in the control, and 3-octen-2-one was only detected in the sterilization groups. Esters are one of the main aromatic compounds with a low flavor threshold, high volatility at room temperature, and good flavor. The relative content of esters in microwave processing was higher than that in stepwise and general retort processing (*P* < 0.05). No acids were detected in the control, microwave, and stepwise retort processing; only 3-hydroxy-dodecanoic acid and 10, 12-octadecadiynoic acid were detected in general retort processing, accounting for only 0.08% of the total volatile compounds. However, as precursors of ester formation, acids also play an important role in the flavor of meat products (43). Xia et al. (44) reported that acids are similar to vinegar and have a low threshold, which results in bad taste for meat products. 2-pentyl-furan and 1-pentyl-1H-pyrrole were only detected for general retort, which belongs to the heterocyclic compounds produced by heat-induced Maillard reaction, with fat, metal odor and low threshold. Shahidi and Oh (45) revealed that 2-pentyl-furan is oxidized by linoleic acid, which is an important factor leading to the formation of a peculiar smell in meat. In addition, 2-pentyl-furan has been listed as a substance that may cause cancer in humans (3). On the whole, the comprehensive aroma performance was in the order of microwave > stepwise retort > general retort.

### Scanning electron microscopy

**FIGURE 3:**
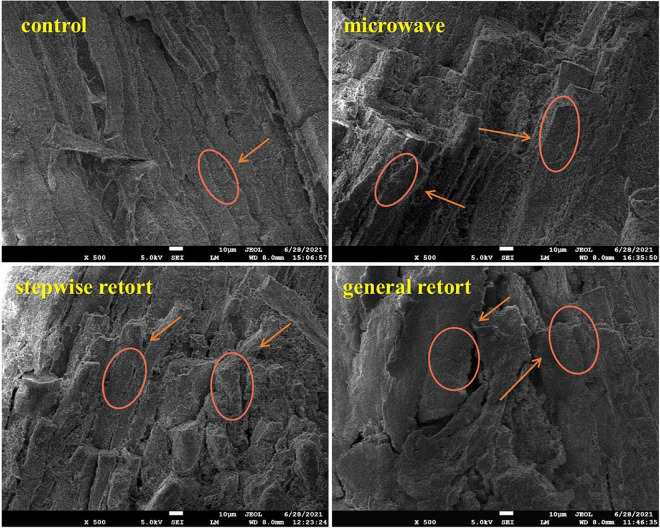
Scanning electron microscopy images of duck meat treated with no thermal sterilization and three sterilization strategies. The circles and arrows indicate the muscle bundle structure of duck meat with different treatments (magnification, 500×).

The microstructure of meat products is closely related to the texture and sensory characteristics of meat products. Figure 3 shows the microstructure of duck meat subjected to the different treatments. According to the arrow and circle areas, different treatments caused different degrees of damage to the microstructure of meat. Compared with control, the degree of damage to muscle bundles in three sterilized groups was in the order of general retort > stepwise retort > microwave. In control, the meat samples had clear and intact muscle bundles with a uniform shape and size; a few muscle bundles were slightly broken; and the boundaries of adjacent muscle bundles were clearly visible and closely arranged. The structure of muscle bundles in microwave and stepwise retort processing was partially broken, but the shape and size of muscle bundles were more uniform. The boundaries of adjacent muscle bundles were clear during microwave processing, whereas the structure of some muscle bundles was disordered, and there were gaps between adjacent muscle bundles in stepwise retort processing. In general, during retort processing, the structure of muscle bundles was seriously damaged; the shape and size were uneven; and the arrangement was irregular. There was a large gap between adjacent muscle bundles, and partial aggregation occurred. This was owing to the fact that heat treatment can aggravate muscle structure destruction and protein accumulation, which was shown in the macroscopic deterioration of the texture of meat products (46). Hence, microwave processing can better retain the microstructure of meat products than stepwise retort processing and general retort processing.

## Conclusion

The quality of unseasoned duck meat after microwave and stepwise retort processing was better than that after general retort processing, with higher water retention, lower loss of nutrients, and better flavor. In order to minimize the quality loss of meat products after thermal sterilization, the microwave parameters and stepwise temperature programs could be optimized. Furthermore, increased efforts should be taken to further improve the quality of duck meat under thermal sterilization by adding seasonings (e.g., sugars, salts, pigments, oils, and amino acids). The results of this study provide a theoretical basis for the application of microwave and stepwise retort technologies in industrial duck meat processing and updating of equipment based on these two technologies.

## Data availability statement

The original contributions presented in this study are included in the article/Supplementary material, further inquiries can be directed to the corresponding author/s.

## Author contributions

XY and YL conducted the experiments, data analyzing, and writing—original draft preparation. PW contributed to the data curation. DL and JS guided the experimental design and reviewed the manuscript before submission. MH and BW conducted the methodology, supervising data, and project administration. YZ conducted the methodology and supervising data. All authors contributed to the article and approved the submitted version.

## References

[1] Wang W Chen H Ke D Chen W Zhong Q Chen W Effect of sterilization and storage on volatile compounds, sensory properties and physicochemical properties of coconut milk. *Microchem J.* (2020) 153:104532. 10.1016/j.microc.2019.104532

[2] Awuah GB Ramaswamy HS Economides A. Thermal processing and quality: principles and overview. *Chem Eng Process.* (2007) 46:584–602. 10.1016/j.cep.2006.08.004

[3] Fardella M Ramírez C Caballero E Sánchez E Pinto M Núñez H Variable retort temperature profiles (VRTPs) and retortable pouches as tools to minimize furan formation in thermally processed food. *Foods.* (2021) 10:2205. 10.3390/foods10092205 34574319 PMC8467077

[4] Luechapattanaporn K Wang Y Wang J Tang J Hallberg LM Dunne CP. Sterilization of scrambled eggs in military polymeric trays by radio frequency energy. *J Food Sci.* (2005) 70:E288–94. 10.1111/j.1365-2621.2005.tb07185.x

[5] Li X Farid M. A review on recent development in non-conventional food sterilization technologies. *J Food Eng.* (2016) 182:33–45. 10.1016/j.jfoodeng.2016.02.026

[6] Sun Y Zhang LL Zhang H Zhang YY Sun BG. Effects of two sterilization methods on the taste compositions of sweet and sour spare ribs flavor. *J Food Composit Anal.* (2021) 104:104143. 10.1016/j.jfca.2021.104143

[7] Tiravibulsin C Lorjaroenphon Y Udompijitkul P Kamonpatana P. Sterilization of coconut milk in flexible packages via ohmic-assisted thermal sterilizer. *LWT.* (2021) 147:111552. 10.1016/j.lwt.2021.111552

[8] Xu J Zhang M Cao P Adhikari B. Effect of ZnO nanoparticles combined radio frequency pasteurization on the protein structure and water state of chicken thigh meat. *LWT.* (2020) 134:110168. 10.1016/j.lwt.2020.110168

[9] Auksornsri T Bornhorst ER Tang J Tang Z Songsermpong S. Developing model food systems with rice based products for microwave assisted thermal sterilization. *LWT.* (2018) 96:551–9. 10.1016/j.lwt.2018.05.054

[10] Wang X Muhoza B Wang X Feng T Xia S Zhang X. Comparison between microwave and traditional water bath cooking on saltiness perception, water distribution and microstructure of grass crap meat. *Food Res Int.* (2019) 125:108521. 10.1016/j.foodres.2019.108521 31554080

[11] Bornhorst ER Liu F Tang J Sablani SS Barbosa-Cánovas GV. Food quality evaluation using model foods: a comparison study between microwave-assisted and conventional thermal pasteurization processes. *Food Bioprocess Technol.* (2017) 10:1248–56. 10.1007/s11947-017-1900-9

[12] Qu Z Tang Z Liu F Sablani SS Ross CF Sankaran S Quality of green beans (*Phaseolus vulgaris* L.) influenced by microwave and hot water pasteurization. *Food Control.* (2021) 124:107936. 10.1016/j.foodcont.2021.107936

[13] Ansorena MR Salvadori VO. Optimization of thermal processing of canned mussels. *Food Sci Technol Int.* (2011) 17:449–58. 10.1177/1082013211398829 21954315

[14] Avila−Gaxiola E Delgado−Vargas F Zazueta−Niebla J López−Angulo G Vega−García M Caro−Corrales J. Variable retort temperature profiles for canned papaya puree. *J Food Process Eng.* (2016) 39:11–8. 10.1111/jfpe.12194

[15] Simpson R Jiménez D Almonacid S Nuñez H Pinto M Ramírez C Assessment and outlook of variable retort temperature profiles for the thermal processing of packaged foods: plant productivity, product quality, and energy consumption. *J Food Eng.* (2020) 275:109839. 10.1016/j.jfoodeng.2019.109839

[16] Cao Z Gao W Zhang Y Huo W Weng K Zhang Y Effect of marketable age on proximate composition and nutritional profile of breast meat from Cherry Valley broiler ducks. *Poult Sci.* (2021) 100:101425. 10.1016/j.psj.2021.101425 34525444 PMC8445895

[17] Guo C Wang Y Luan D. Non-thermal effects of microwave processing on inactivation of *Clostridium Sporogenes* inoculated in salmon fillets. *LWT.* (2020) 133:109861. 10.1016/j.lwt.2020.109861

[18] Huo W Weng K Gu T Zhang Y Zhang Y Chen G Effect of muscle fiber characteristics on meat quality in fast- and slow-growing ducks. *Poult Sci.* (2021) 100:101264. 10.1016/j.psj.2021.101264 34174572 PMC8242056

[19] Jo Y An KA Arshad MS Kwon JH. Effects of e-beam irradiation on amino acids, fatty acids, and volatiles of smoked duck meat during storage. *Innov Food Sci Emerg Technol.* (2018) 47:101–9. 10.1016/j.ifset.2017.12.008

[20] Hwang SI Lee EJ Hong GP. Effects of temperature and time on the cookery properties of sous-vide processed pork loin. *Food Sci Anim Resour.* (2019) 39:65–72. 10.5851/kosfa.2019.e4 30882075 PMC6411237

[21] Jin S Pang Q Yang H Diao X Shan A Feng X. Effects of dietary resveratrol supplementation on the chemical composition, oxidative stability and meat quality of ducks (*Anas platyrhynchos*). *Food Chem.* (2021) 363:130263. 10.1016/j.foodchem.2021.130263 34116495

[22] Rao JW Meng FB Li YC Chen WJ Liu DY Zhang JM. Effect of cooking methods on the edible, nutritive qualities and volatile flavor compounds of rabbit meat. *J Sci Food Agric.* (2022) 102:4218–28. 10.1002/jsfa.11773 35038172

[23] Cai L Feng J Cao A Zhang Y Lv Y Li J. Denaturation kinetics and aggregation mechanism of the sarcoplasmic and myofibril proteins from grass carp during microwave processing. *Food Bioproc Technol.* (2018) 11:417–26. 10.1007/s11947-017-2025-x

[24] Choi JI Kim JK Kim JH Kweon DK Lee JW. Degradation of hyaluronic acid powder by electron beam irradiation, gamma ray irradiation, microwave irradiation and thermal treatment: a comparative study. *Carbohydr Polym.* (2010) 79:1080–5. 10.1016/j.carbpol.2009.10.041

[25] Khan MA Ali S Abid M Ahmad H Zhang L Tume RK Enhanced texture, yield and safety of a ready-to-eat salted duck meat product using a high pressure-heat process. *Innov Food Sci Emerg Technol.* (2014) 21:50–7. 10.1016/j.ifset.2013.10.008

[26] Li C Wang D Xu W Gao F Zhou G. Effect of final cooked temperature on tenderness, protein solubility and microstructure of duck breast muscle. *LWT.* (2013) 51:266–74. 10.1016/j.lwt.2012.10.003

[27] Wang Q Jiao X Yan B Meng L Cao H Huang J Inhibitory effect of microwave heating on cathepsin L-induced degradation of myofibrillar protein gel. *Food Chem.* (2021) 357:129745. 10.1016/j.foodchem.2021.129745 33894571

[28] Mehmood W Qian S Zhang C Li X. Biophysical properties and volumetric changes in breast meat of broilers and yellow-feathered chicken as affected by cooking process. *Int J Food Prop.* (2019) 22:1935–51. 10.1080/10942912.2019.1696361

[29] Stangierski J Tomaszewska-Gras J Baranowska HM Krzywdzińska-Bartkowiak M Konieczny P. The effect of deep pectoral myopathy on the properties of broiler chicken muscles characterised by selected instrumental techniques. *Eur Food Res Technol.* (2019) 245:459–67. 10.1007/s00217-018-3177-2

[30] Cao C Xiao Z Tong H Tao X Gu D Wu Y Effect of ultrasound-assisted enzyme treatment on the quality of chicken breast meat. *Food Bioprod Process.* (2021) 125:193–203. 10.1016/j.fbp.2020.11.005

[31] Khan MA Ali S Yang H Kamboh AA Ahmad Z Tume RK Improvement of color, texture and food safety of ready-to-eat high pressure-heat treated duck breast. *Food Chem.* (2019) 277:646–54. 10.1016/j.foodchem.2018.11.006 30502199

[32] Chen X Luo J Lou A Wang Y Yang D Shen QW. Duck breast muscle proteins, free fatty acids and volatile compounds as affected by curing methods. *Food Chem.* (2021) 338:128138. 10.1016/j.foodchem.2020.128138 33091978

[33] Calabrò E Magazù S. Non-thermal effects of microwave oven heating on ground beef meat studied in the mid-infrared region by fourier transform infrared spectroscopy. *Spectrosc Lett.* (2014) 47:649–56. 10.1080/00387010.2013.828313

[34] Lucarini M Durazzo A Del Pulgar JS Gabrielli P Lombardi-Boccia G. Determination of fatty acid content in meat and meat products: the FTIR-ATR approach. *Food Chem.* (2018) 267:223–30. 10.1016/j.foodchem.2017.11.042 29934161

[35] Calabrò E Magazù S. Modulation of Maillard reaction and protein aggregation in bovine meat following exposure to microwave heating and possible impact on digestive processes: an FTIR spectroscopy study. *Electromagn Biol Med.* (2020) 39:129–38. 10.1080/15368378.2020.1737805 32142387

[36] Yu Q Fang C Ma Y He S Ajuwon KM He J. Dietary resveratrol supplement improves carcass traits and meat quality of Pekin ducks. *Poult Sci.* (2021) 100:100802. 10.1016/j.psj.2020.10.056 33518308 PMC7936143

[37] Moran L Aldai N Barron LJR. Elucidating the combined effect of sample preparation and solid-phase microextraction conditions on the volatile composition of cooked meat analyzed by capillary gas chromatography coupled with mass spectrometry. *Food Chem.* (2021) 352:129380. 10.1016/j.foodchem.2021.129380 33667923

[38] Huang Q Dong K Wang Q Huang X Wang G An F Changes in volatile flavor of yak meat during oxidation based on multi-omics. *Food Chem.* (2022) 371:131103. 10.1016/j.foodchem.2021.131103 34537608

[39] Yuan X Zhu X Sun R Jiang W Zhang D Liu H Sensory attributes and characterization of aroma profiles of fermented sausages based on fibrous-like meat substitute from soybean protein and *Coprinus comatus*. *Food Chem.* (2022) 373:131537. 10.1016/j.foodchem.2021.131537 34776312

[40] Li X Xie W Bai F Wang J Zhou X Gao R Influence of thermal processing on flavor and sensory profile of sturgeon meat. *Food Chem.* (2022) 374:131689. 10.1016/j.foodchem.2021.131689 34875433

[41] Xie J Sun B Zheng F Wang S. Volatile flavor constituents in roasted pork of Mini-pig. *Food Chem.* (2008) 109:506–14. 10.1016/j.foodchem.2007.12.074

[42] Sicuro B. The future of caviar production on the light of social changes: a new dawn for caviar? *Rev Aquac.* (2019) 11:204–19. 10.1111/raq.12235

[43] Ansorena D Gimeno O Astiasaran I Bello J. Analysis of volatile compounds by GC–MS of a dry fermented sausage: chorizo de Pamplona. *Food Res Int.* (2001) 34:67–75. 10.1016/S0963-9969(00)00133-2

[44] Xia C He Y Cheng S He J Pan D Cao J Free fatty acids responsible for characteristic aroma in various sauced-ducks. *Food Chem.* (2021) 343:128493. 10.1016/j.foodchem.2020.128493 33158671

[45] Shahidi F Oh WY. Lipid-derived flavor and off-flavor of traditional and functional foods: an overview. *J Food Bioact.* (2020) 10:20–31. 10.31665/JFB.2020.10224

[46] Islam MN Zhang M Adhikari B Xinfeng C Xu BG. The effect of ultrasound-assisted immersion freezing on selected physicochemical properties of mushrooms. *Int J Refrig.* (2014) 42:121–33. 10.1016/j.ijrefrig.2014.02.012

