# Supplementary material for: Quality changes of duck meat during thermal sterilization processing caused by microwave, stepwise retort, and general retort heating
Source: Front Nutr. 2022 Oct 20;9:1016942. doi: 10.3389/fnut.2022.1016942 (PMC9630348; doi:10.3389/fnut.2022.1016942)
Supplement: Supplementary file 1 [file Data_Sheet_1.PDF]

Supplementary Materials

1 Sensory evaluation

1.1 Method

The duck breast meat treated with different sterilization was evaluated by 8 trained sensory experts in terms of color, smell, taste, tissue status and overall acceptability. The score of each index ranges from 1 (very poor) to 9 (very good).

1.2 Results and discussion

Table S1 shows the effects of different sterilization treatments on the sensory quality of duck meat. The results showed that there was no significant difference ($P > 0.05$) in color score among the groups. The scores of smell, taste, tissue states and overall acceptability of duck meat treated by general retort were significantly lower than those of control ($P < 0.05$), while the scores of smell, taste and overall acceptability of duck meat treated by microwave and stepwise retort were not significantly different ($P > 0.05$) from those of control. This indicates that the sterilization treatment reduces the sensory quality of duck meat, and consumers have higher acceptance of duck meat treated with microwave and stepwise retort among the three sterilization treatments. In terms of tissue status, the score of the duck meat after sterilization was significantly lower than that of control ($P < 0.05$), and the tissue status of microwave group was better in the sterilization groups, which was consistent with the determination of the texture properties. Overall, the results of sensory evaluation of microwave and stepwise retort groups were better.

Table S1 Sensory evaluation of duck meat treated with no thermal sterilization and three sterilization strategies

Treatment	Color	Smell	Taste	Tissue status	Overall acceptability
control	6.25 ± 1.04^{a}	7.25 ± 1.16^{a}	7.63 ± 0.92^{a}	8.38 ± 0.74^{a}	7.38 ± 0.92^{a}
microwave	7.25 ± 1.28^{a}	6.50 ± 1.20^{ab}	7.50 ± 0.93^{a}	7.00 ± 0.93^{b}	6.75 ± 1.16^{ab}
stepwise retort	6.88 ± 1.13^{a}	6.63 ± 0.92^{ab}	7.00 ± 1.31^{ab}	6.13 ± 1.36^{bc}	6.63 ± 0.92^{ab}
general retort	6.38 ± 1.19^{a}	5.50 ± 0.53^{b}	5.75 ± 1.04^{b}	5.25 ± 1.04^{c}	5.75 ± 1.04^{b}

The values are expressed as mean ± standard deviation (n = 3). Different lowercase letters (a-c) in the same column represent significant differences ($P < 0.05$).
